# Enhancing Plasticity of the Central Nervous System: Drugs, Stem Cell Therapy, and Neuro-Implants

**DOI:** 10.1155/2017/2545736

**Published:** 2017-12-17

**Authors:** Alice Le Friec, Anne-Sophie Salabert, Carole Davoust, Boris Demain, Christophe Vieu, Laurence Vaysse, Pierre Payoux, Isabelle Loubinoux

**Affiliations:** ^1^ToNIC, Toulouse NeuroImaging Center, Université de Toulouse, Inserm, UPS, Toulouse, France; ^2^Radiopharmacy Department, CHU Toulouse, Toulouse, France; ^3^LAAS-CNRS, Université de Toulouse, CNRS, INSA, UPS, Toulouse, France; ^4^Nuclear Medicine Department, CHU Toulouse, Toulouse, France

## Abstract

Stroke represents the first cause of adult acquired disability. Spontaneous recovery, dependent on endogenous neurogenesis, allows for limited recovery in 50% of patients who remain functionally dependent despite physiotherapy. Here, we propose a review of novel drug therapies with strong potential in the clinic. We will also discuss new avenues of stem cell therapy in patients with a cerebral lesion. A promising future for the development of efficient drugs to enhance functional recovery after stroke seems evident. These drugs will have to prove their efficacy also in severely affected patients. The efficacy of stem cell engraftment has been demonstrated but will have to prove its potential in restoring tissue function for the massive brain lesions that are most debilitating. New answers may lay in biomaterials, a steadily growing field. Biomaterials should ideally resemble lesioned brain structures in architecture and must be proven to increase functional reconnections within host tissue before clinical testing.

## 1. Introduction

Pathologies such as stroke remain chronically debilitating despite scientific advances in the vast field of CNS injury. Following the acute phase, there are no effective treatments available to patients besides physiotherapy.

It is now well known that various mechanisms of brain plasticity occur after stroke onset, both in the acute phase and beyond [1–6]. They may partially account for the spontaneous recovery of motor function [7]. Therefore, drug treatments have increasingly aimed to enhance these processes in order to improve functional recovery [8].

As for tissue repair of the lesioned area, endogenous neurogenesis does not however produce mature neuronal and glial cells in a sufficient number to completely regenerate lesioned CNS tissue [9]. Over the last decades, this observation has led to intense focus on stem cell therapy for the treatment of acute and focal CNS damage produced by pathologies such as stroke, traumatic brain injury, and spinal cord injury (SCI). Transplanted stem cells are expected to (i) exert trophic effects on host tissue by secretion of beneficial factors and/or (ii) actually replace lost tissue and establish functional short- or long-distance connections with host cells. Numerous neural and nonneural stem cell types have shown promise in experimental rodent models of stroke [10, 11] and nonhuman primate (NHP) models of SCI [12]. This preclinical evidence has allowed stem cell delivery to be clinically tested for safety and efficacy in the treatment of stroke [13, 14], TBI [15, 16], and SCI [17]. However, stem cell trials for brain repair have yet to show consistent results respective to efficacy and functional improvement in man [18].

Indeed, when considering stem cell graft within the lesion site, it is important to stress the inhospitable nature of the tissue. Excitotoxicity, inflammatory processes, glial scar formation, growth-inhibiting factors, abnormal tissue structure, and loss of extracellular matrix components render the lesion site unfavorable to neuroblast survival and differentiation [19, 20]. Stem cells grafted close to the brain lesion may die despite immunosuppressant therapy [21].

A promising way to provide endogenous neuroblasts and grafted cells with a suitable microenvironment may consist in the development of biomaterial ECM replacements and “scaffolds” [22]. Biomaterials aiming to mimic the ECM have enhanced tissue reconstruction in models of stroke [23]. They may also be engineered to deliver trophic factors [24] or to guide axonal growth [25]. Implantation of biomaterial has just reached first-in-man clinical testing in the injured spinal cord [26].

Cotransplantation of biomaterial and stem cells has been successfully tested in preclinical studies for the treatment of stroke in the chronic phase in rodents [27, 28]. Although the translation of such therapies to the clinic presents technical challenges, we believe this technology opens up exciting avenues of treatment for focal chronic brain injury.

Here, we propose to review the most recent innovative drug-, stem cell-, and biomaterial-based therapies for the treatment of CNS injuries such as those caused by stroke and SCI.

### 1.1. Drugs

#### 1.1.1. Drugs for Axon Repair

Central nervous system axons, unlike those in the peripheral nervous system, were long thought to have lost their capacity for regeneration after being sectioned. This concept now seems outdated. Many recent studies have revealed the existence of proteins, such as NOGO, within the myelin sheath that are capable of inhibiting axonal growth and preventing axonal regeneration after a lesion. Drugs targeting these inhibitory proteins, such as anti-NOGOs, have been successfully tested in rodents and primates. Cramer et al. conducted a double-blinded placebo-controlled pilot study of GSK249320, a monoclonal anti-MAG (myelin-associated glycoprotein) antibody, in patients presenting a moderate walking disability after stroke (0.5 m/sec on average 5 days after stroke). The drug was administered 24 h and 9 days after the stroke onset and was well tolerated at the three doses tested (1, 5, or 15 mg/kg, i.v.). Only the 5 mg/kg (*n* = 9) dose significantly improved walking speed against placebo (*n* = 17) in a 112-day period, and recovery was particularly marked in the first 60 days [29]. This result suggests that dose and duration of treatment may be further optimized. Experimental testing in animals also showed that early administration within the first week may be more efficient [30]. Unfortunately, a recent large trial on 134 patients was interrupted for lack of efficacy despite the safety of the humanized monoclonal antibody [31]. However, anti-NOGO or other molecules may prove the efficacy of this strategy in the future.

#### 1.1.2. Growth Factors

Growth factors such as G-CSF (granulocyte colony-stimulating factor), known to recruit hematopoietic stem cells, have been considered for use in stroke therapy based on the rationale that they possess such beneficial properties in the acute phase of stroke such as the inhibition of glutamate secretion, reduction of inflammation, and antiapoptotic and antiedema effects, as well as proangiogenesis and neurogenesis properties in the chronic phase [32]. However, no functional improvement was evidenced in a cohort of 548 patients [33]. Similar results were found for other growth factors, such as bFGF (basic fibroblast growth factor or trafermin), known to increase neurite growth. When administered in the acute phase, bFGF caused systemic adverse effects and mortality. The phase II/III trial was interrupted at 286 patients [34]. Another neurotrophic factor, brain-derived neurotrophic factor, was shown to be toxic. Thus, it is not currently feasible to consider the use of such growth factors for therapy after ischemic stroke.

#### 1.1.3. Selective Serotonin Reuptake Inhibitors (SSRIs)

Our team in Toulouse has focused on neuroimaging as a means to develop and adapt biomarker-based therapeutic strategies. We propose candidate biomarkers (1) for the use in motor outcome prediction [35–37] and (2) as therapeutic agents with proven efficacy as evaluated by fMRI [38–44]. Our work, which was confirmed by other teams, has demonstrated that the ipsilesional motor cortex M1 is a key structure of motor recovery and is thus a suitable target for drug-, stem cell-, and noninvasive brain stimulation-based therapies. Functional activations in the primary sensorimotor cortex may be enhanced by the administration of monoaminergic drugs. Drug-induced hyperactivations have been positively correlated with motor improvement, even in unique doses of treatment. However, this result was elicited in small groups of moderately disabled stroke patients, and work must be extended to more severely affected patients, who respond modestly to interventions. Our group demonstrated, in a double-blind placebo-controlled multicentric clinical trial of 118 patients, including heavily affected stroke patients, that fluoxetine (Prozac) treatment significantly improves motor recovery (Fugl-Meyer scale and motor NIHSS) when compared to placebo. Functional improvement was observed, and a higher number of patients regained independence in the treatment group (modified Rankin Score (mRS)) [45]. In a recent study with another SSRI, a similar result was found along with a 50% reduction in the 3-month National Institutes of Health Stroke Scale compared with the baseline scores. This was achieved in 57 patients on the citalopram and 39 patients on the placebo group [46]. Recommendations for the design of clinical drug studies in stroke have been produced [47]. The Cochrane review reported that while SSRIs may improve patient independence, deficit, and neurological status, as well as lessening anxiety and depression, intertrial heterogeneity limits the drawing of meaningful conclusions. Larger clinical trials are needed to validate fluoxetine as stroke treatment before it can be prescribed routinely in the clinic [48] and must confirm the treatment efficacy and determine the optimal dose and length of treatment. To this end, phase III trials have been launched in Australia (http://affinitytrial.org), Sweden (http://www.effects.se), and the United Kingdom (http://focustrial.org.uk) [49] and aim to include 6000 patients, 4530 of which have already been enrolled (FOCUS 3127 (closed), AFFINITY 522, and EFFECT 881). SSRIs induce only minor and well-known adverse effects and are well tolerated in stroke patients. Although clinical evidence of efficacy is pending, the benefit-to-risk ratio seems for now in favor of SSRIs' prescription after ischemic stroke.

When considering the mechanism of action of this antidepressant, it is useful to evoke the historic experiments that first evidenced the concomitant firing of neurons in the raphe nucleus during movement and increased synaptic strength of the sensorimotor synapse and short-term and long-term facilitation, leading Jacobs and Fornal to propose motor facilitation as a primary function of the serotoninergic system [50]. It follows that the benefit of SSRI treatment may be further enhanced by physiotherapy. Furthermore, recent studies have described other biological effects of SSRI drugs such as anti-inflammatory properties through microglial repression and reduction of neutrophil infiltration [51, 52], an increase in BDNF secretion [53], and enhancement of neurogenesis (see the next section) and neural stem cell survival and differentiation [54, 55], even in aged-brain lesioned rats [56]. In line with the neurogenic effect of SSRI, studies have shown that fluoxetine improves declarative memory and increases hippocampal volume in patients suffering from a posttraumatic stress disorder [57, 58].

### 1.2. Stem Cell Engraftment

Neurogenesis, defined as the capacity of the brain to produce new neurons, has been evidenced in man [59] in neurogenic brain regions, namely, the dentate gyrus of the hippocampus and in the subventricular zone of the cortex. These niches produce stem cells and progenitor cells that are capable of migrating to damaged cortical and/or subcortical brain areas and replacing lost neurons in patients after stroke [1, 9, 60, 61]. However, few neuroblasts survive to reach full neuronal differentiation. Those that do often remain confined to the lesion border and are thus incapable of replacing extensive losses of neuronal tissue. Recent work has shown that as few as 0.2% of lost neurons are replaced [9].

Stem cell-based therapeutic strategies aim to support and/or stimulate endogenous neurogenesis by engraftment of stem cells, most often through intravenous or intracerebral delivery. One benefit of stem cell therapy may be the release of neuroprotective, trophic, or immunomodulatory factors by grafted cells. These so-called trophic effects occur rapidly after engraftment and may stimulate endogenous neurogenesis, angiogenesis, and neovascularization, as well as reducing apoptosis and inflammation [62]. However, for massive brain injury and severely affected patients, trophic effects will unlikely allow sufficient tissue regeneration. In these cases particularly, engraftment of stem cells with a view to not only provide trophic support but also replace damaged neurons and brain tissue could be considered.

The least invasive method of stem cell delivery remains the intravenous method. This procedure is carried out for the delivery of hematopoietic or mesenchymal stem cells. Clinical trials must meet stringent GMP (good manufacturing practice) norms that regulate the quality and safety of cells for engraftment. These regulations dictate all aspects of cell origin, from the composition of cell culture mediums (which should avoid reliance on products of animal origin) to the cell banks from which the cells are selected, which must be genetically stable and homogenous and regularly tested for identity, viability, and sterility.

#### 1.2.1. Mesenchymal Stem Cells

*(1) Intravenous Delivery*. Mesenchymal stem cells have the advantage of being relatively easy to isolate and amplify from readily accessible tissue samples. In particular, they may be extracted more easily from fat tissue than from bone marrow. Allogenic stem cell transplantation is rendered possible by the fact that these cells do not express the major histocompatibility complex (MHC) antigen. Mesenchymal stem cells can be differentiated into many cell types (chondrocytes, osteoblasts, osteocytes, adipocytes, myocytes, and tendinocytes) and possess capacity for migration toward damaged tissue in the brain [63]. Intravenous administration of adult mesenchymal stem cells has been proven safe thus far [64–66] and potentially efficient. A recent study found that intravenous delivery of multipotent progenitor cells, although well tolerated, did not produce significant improvement [67]. However, the number of patients included (*n* = 126, intent-to-treat population) may not have provided sufficient statistical power to show modest effects. Clinical trials to evaluate the efficacy of the approach are ongoing. It is likely that any beneficial properties will result from trophic effects, which may reduce neuroinflammation in the acute phase and support the neovascularization within the damaged parenchyma.

*(2) Intracerebral Delivery*. A recent phase I/2a American trial has demonstrated the safety of an intracerebral graft of mesenchymal stem cells, genetically engineered to transiently express notch-1, a factor known to drive neuronal differentiation [13]. 18 patients with ischemic brain damage (11 of whom were women), with an average age of 61 years, and presenting a stable and chronic motor deficit received the graft between 6 and 20 months after injury and were followed for a year (*n* = 16). 2.5, 5, or 10 million SB263 cells produced by SanBio were injected into the peri-infarct. Proof-of-concept research showed cell survival 1 month after transplantation in cerebrolesioned animals [13]. One serious adverse event was declared (asymptomatic subdural hematoma). NIHSS neurological scale, European stroke scale, and Fugl-Meyer scale results evidenced significant improvement of recovery in graft recipients. However, for ethical reasons, this study was not controlled by a group of patients receiving a control surgical procedure.

#### 1.2.2. Intraspinal Graft of Olfactory Ensheathing Stem Cells

Autologous engraftment of olfactory ensheathing cells, harvested from the olfactory mucosa of 3 chronic medullar injury patients, produced a quite spectacular improvement in American Spinal Injury Association class (A to B or C) scores in two patients and more local enhancement of motricity and sensitivity in the third patient [17]. Though the mechanisms of action of these cells are far from elucidated, it has been suggested that these “support cells” may reduce glial scar formation, rendering the lesion site more permissive to axonal regeneration.

#### 1.2.3. Intracerebral Graft of Neural Stem Cells

The main challenge in tissue regeneration therapies is not only the replacement of lost neurons but also the establishment of functional reconnections. In this view, selecting a cell source is difficult.

In the first phase 2 randomized clinical trial led by Kondziolka et al., the feasibility of intracerebral stem cell engraftment in 14 stable stroke patients was demonstrated [68, 69]. Although the hNT2 (LBS-Neurons, Layton Bioscience) stem cell line was successfully differentiated into neurons, this line originates from a teratocarcinoma which is no longer authorized for trial in man due to its extremely abnormal karyotype. The study included a small (*n* = 4) group of control patients, paired for physiotherapy. Six out of eleven PET scans evidenced an improvement of glucose intake at the implantation site (3 injections were performed: above, within, and below the lesion site). Improvement of functional recovery was not significant in the treated group compared to controls. Four treated patients, who presented lesions in the nondominant hemisphere, showed enhanced performance in the figure of Rey test. This suggests improved visuospatial skills and nonverbal memory [70].

A recent phase 1 first-in-man study used the CTX0E03 or ReN001 cell line (ReNeuron) derived from genetically modified embryonic stem cells originating from the human fetal neuroepithelium [14]. In order to control the amplification of cells, they used c-mycERT AM technology to drive expression of an oestradiol receptor under tamoxifen (4-OHT) induction (added to culture medium). Cell division is arrested, and differentiation into neuronal and glial lineages was induced by removal of tamoxifen and growth factors from the medium. It is important to note that the use of tamoxifen for the treatment of breast cancer in women could restart division of the transplanted cells. For this reason, women were excluded from the protocol. Eleven men presenting a moderate-to-severe disability were enrolled for perilesional grafting of 2, 5, 10, or 20 million cells 6 to 60 months after the stroke onset. Patients did not receive any immunosuppressive therapy. Patients were followed for 2 years as part of this noncontrolled trial. No immunological or adverse effects were attributed to the grafted cells. Modest improvements of different motor scales were observed (NIHSS, Barthel index, Ashworth Spasticity Scale for the arm and leg, and a quality of life and health status EuroQoL Five Dimensions questionnaire EQ-5D).

Although the setup of methodologies to control trials with groups of operated-upon but nongrafted patients poses for now unsurmountable technical and ethical difficulties, the true efficacy of stem cell-based interventions cannot be fully validated without this condition and larger patient cohorts. Perilesional injection of cells into healthy tissue is often performed in order to optimize stem cell survival. The rapidly occurring trophic effects of this approach are now well established; however, true functional replacement of lost cells remains to be solidly demonstrated although difficult to test in humans.

While regenerative medicine strategies aim to replace the lesioned neural tissue by intracerebral engraftment, the lesion site microenvironment is unconducive to progenitor survival and differentiation due to the destruction of extracellular matrix (ECM) components which is replaced or isolated by scar tissue [19, 71]. Effectiveness of therapy is limited as only 5% of grafted cells survive. An exciting solution to this problem may be produced by nanotechnology scaffolds.

### 1.3. Neuro-Implants

Biomaterials may provide a suitable support for cells, replacing the lost extracellular matrix. They may promote cell survival and differentiation, revascularisation, and recolonisation of the lesioned tissue by glial and endothelium cells from the host. More complex biomimetic materials may also guide axonal growth towards their biological targets, restoring effective and even long-distance connections between damaged and healthy tissues. Where stroke is concerned, research in this innovative field remains currently preclinical.

#### 1.3.1. Injectable Nanometric Biomaterials

*(1) Nanofibers*. Fibrous biomaterials of nanometric dimension were injected in scar tissue in a rat model of medullar lesion. They were composed of peptides that autoassemble to form fibers and contain epitopes of laminin, an ECM component involved in processes such as cell adhesion. Axons of the descending corticospinal tract and those of the ascending sensory neurons that could not previously cross the fibrous glial scar were able to penetrate the biomaterial and cross the lesion. Importantly, motor recovery was significantly enhanced in treated animals [72]. A biodegradable and biocompatible block copolymer of poly-lactic-co-glycolic acid and poly-L-lysine improves functional recovery of rats and nonhuman primates after a partial and complete lateral hemisection of the thoracic spinal cord [73]. INSPIRE, a clinical trial, is ongoing, and the safety of this approach in man has been published in one case [26].

*(2) Hydrogels*. Polymer hydrogels are another candidate biomaterial for the support of grafted cells. For instance, polyglycolic acid (PGA) is often used as it is porous, biodegradable, and entirely synthetic, meaning its exact composition can be easily controlled. Park et al. included neural stem cells in a soluble hydrogel which then polymerizes within the lesion site [74]. They demonstrated convincing tissue reconstruction in a rodent model of ischemic stroke (middle cerebral artery occlusion (MCAo)) which produces massive lesions. The biomaterial is conducive to neurite growth, and connections were evidenced between the host and grafted cells. Vascularisation and reduction of the glial scar and of monocyte infiltration were also found. This type of approach has shown promising results for sensorimotor and cognitive recovery [75].

#### 1.3.2. Micrometric Injectable Biomaterials

*(1) Microbeads*. Easily injectable micrometric biomaterial beads have also been developed. When injected in a rat model of Parkinson disease, they improved motricity, decreased striatal lesion volume, and reduced substantia nigra degeneration [76].

**Figure 1:**
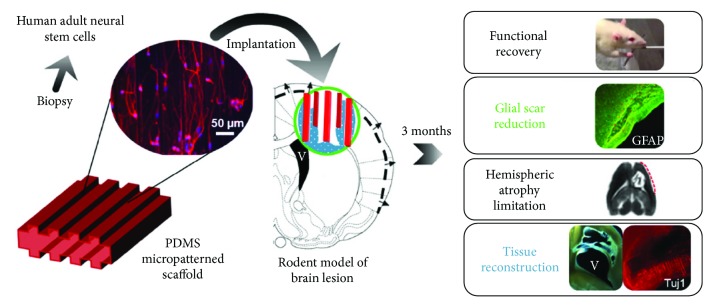
Neuro-implant concept. Guiding scaffolds located in the lesion of the corticospinal tract may improve tissue reconstruction and appropriate direction of regenerated tracts.

**Figure 2:**
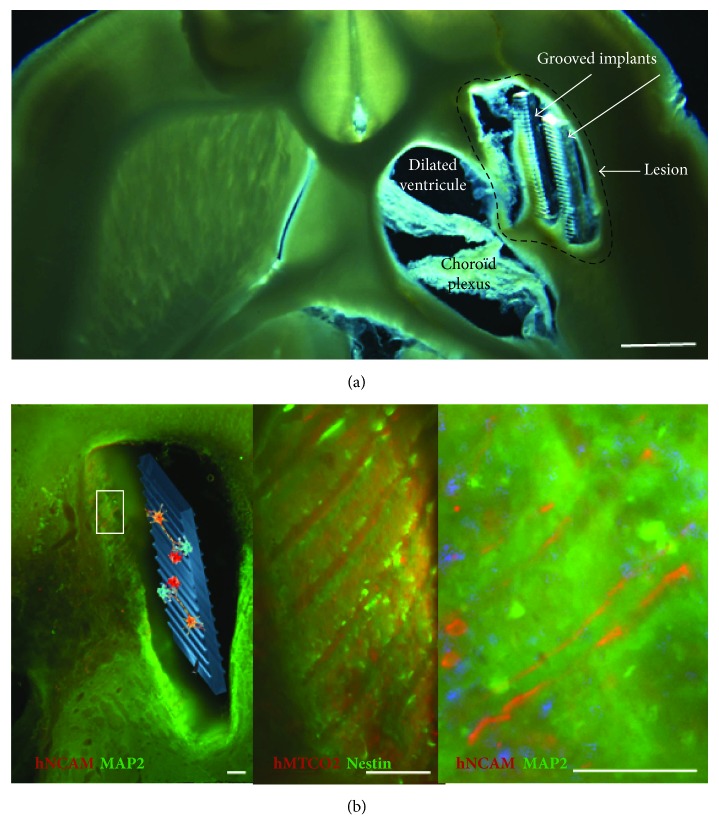
Representative horizontal brain section of the lesioned area of rats with implants alone (a) (scale bar: 1 mm) and neuro-implants (b) under brightfield illumination. The newly generated tissue was mostly located around the PDMS implants. (b) Human neural stem cells were identified by a specific human marker hNCAM or hMTCO2, in combination with a marker (in green) of immature (nestin) and mature (MAP2) neurons. Low magnification is provided on the left and higher magnifications on the right (scale bars: 100 *μ*m). Grafted cell neurites were aligned along the grooves of the implant.

**Figure 3:**
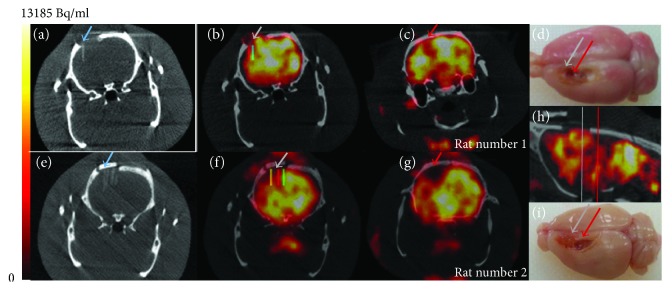
Measurement of cerebral blood flow by nanoSPECT Plus-CT Bioscan with [^99m^Tc]-HMPAO. Fifteen minutes after intravenous injection of 50 MBq of [^99m^Tc]-HMPAO in the tail vein of Sprague-Dawley anesthetized rats, data were acquired during 7 min for SPECT (48 sec and 100000 cps per projection, image size 276 × 276 × 164, 0.1 mm) and 1 min for CT (55 kVp, 500 msec, pitch 0.5, binning 1 : 4). Following the reconstruction, the CT images were spatially aligned to match the SPECT images. Processing of reconstructed images was performed with the in-house Sysiphe software [79]. Brain implants were identified on CT (blue arrows), and 3D volumes of interest (VOIs) were drawn on either side of the implants (colored rectangles) and symmetric ROIs were drawn on the contralateral side as a control (not shown). Images of two rats 20 days after a corticostriatal lesion and 7 days after implantation of neuro-implants. (a, e) CT scan of the brain implants (blue arrows). One implant was inserted in rat number 1 brain and 5 implants in rat number 2 brain. (b, f) SPECT-CT with HMPAO radiotracer on the area of the brain implant. (c, g) SPECT-CT with HMPAO radiotracer on the area of brain damage (located behind the implantation zone). We observed major hypoperfusion (red arrow). The presence of implants limited the hypoperfusion: for rat number 1, −13% in (b) compared to −25% in (c) (ROI volume was 0.4 mm^3^) and for rat number 2, −18% in (f) compared to −57% in (g) (ROI volume was 1.5 mm^3^). (h) Sagittal view of rat number 1. Coronal views (b, c) are located with grey and red lines. (d, i) Rat brain perfused and extracted 3 months after the lesion showing the lesion area where neuro-implants were inserted (grey arrows) or not (red arrows).

*(2) Structured and Guiding Biomaterial Implants*. Our team has proposed a strategy for the long-distance bridging of brain regions using biomaterials seeded with neural stem cells, called neuro-implants, in collaboration with LAAS-CNRS (Figure 1). They are made with PDMS (polydimethylsiloxane) and microstructured to guide axonal growth in predefined directions (Figure 2). We have conducted a proof-of-concept study of the efficacy of neuro-implants compared to implants alone in a rat model of corticostriatal lesion impacting the corticospinal tract, which produces loss of forelimb strength and dexterity [77]. The implants did not increase reactive astrogliosis, scarring, or inflammatory responses. They improved the survival of grafted cells, their maturation, and partial tissue reconstruction within the lesion site around the implants. Reconstructed tissue around the neuro-implants was vascularized as assessed by the HMPAO radiotracer perfusion with SPECT imaging (Figure 3). In contrast, lesioned tissue without implants evolved in a cystic cavity (Figure 3, red arrows). The increase in number of surviving grafted cells may also have trophic effects on cerebral plasticity, such as growth factor and anti-inflammatory factor secretion [78].

## 2. Conclusion

In summary, effective drug therapies are gradually becoming available to improve functional recovery after stroke. However, these will unlikely allow spectacular gains in patients with severe brain damage. Many research teams currently strive to demonstrate the efficacy of stem cell transplantation, which has shown promise in many preclinical models of brain injury. Nonetheless, stem cells alone may not repair the most extensive and debilitating lesions. Much hope has arisen from the development of biomaterial scaffolds, a rapidly growing field of research. These would ideally resemble the architecture of the brain in structure [80] and be proven to allow adequate reconnections with host tissue if possible. If not, given the complexity of this approach, they must at least provide a very high benefit before they can be considered in a clinical setting.
